# Thoracoscopic one-stage lobectomy and diaphragmatic plication for T3 lung cancer

**DOI:** 10.1186/s13019-018-0766-x

**Published:** 2018-07-09

**Authors:** Yuki Takahashi, Masahiro Miyajima, Taijiro Mishina, Ryunosuke Maki, Makoto Tada, Kodai Tsuruta, Atsushi Watanabe

**Affiliations:** Department of Thoracic Surgery, Sapporo Medical University, School of Medicine and Hospital, South 1, West 16, Chuo-ku, Sapporo, Hokkaido 060-8556 Japan

**Keywords:** Thoracoscopic lobectomy, Phrenic nerve resection, Diaphragmatic paralysis, Diaphragmatic plication, One-stage surgery

## Abstract

**Background:**

Combined resection of a phrenic nerve is occasionally required in T3 primary lung carcinomas invading the phrenic nerve to completely remove a malignant tumour, resulting in diaphragmatic paralysis. We describe the first case of thoracoscopic lobectomy and diaphragmatic plication as a one-stage surgery for lung cancer invading the phrenic nerve.

**Case presentation:**

A 56-year-old woman with a T3N0M0 primary adenosquamous carcinoma in the left upper lobe presented with suspicious invasion to the anterior mediastinal fat tissue and left phrenic nerve and underwent left upper lobectomy, node dissection, and partial resection of the anterior mediastinal fat tissue with the left phrenic nerve. Furthermore, thoracoscopic diaphragmatic plication was performed as a concomitant procedure. The patient’s postoperative course was favourable, without any complications, and respiratory function was preserved for 1 year postoperatively.

**Conclusions:**

Thoracoscopic one-stage lobectomy and diaphragmatic plication for T3 lung cancer invading the phrenic nerve is effective for preservation of postoperative pulmonary function.

## Background

Combined resection of the phrenic nerve is occasionally required in T3 primary lung carcinomas invading the nerve to completely remove a malignant tumour. This can cause diaphragmatic paralysis. Postoperative diaphragmatic paralysis leads to impaired respiratory function and pulmonary complications [1]. Diaphragmatic plication has been performed for the surgical treatment of diaphragmatic paralysis and eventration. Tokunaga et al. demonstrated the efficacy of intraoperative diaphragmatic plication by comparing postoperative vital capacity (VC) and forced expiratory volume in 1 s (FEV1) with predicted postoperative VC (ppo VC) and predicted postoperative FEV1 (ppo FEV1) for patients undergoing unilateral phrenectomy during extended surgery [2]. However, thoracoscopic one-stage pulmonary lobectomy, partial resection of the phrenic nerve, and diaphragmatic plication have seldom been performed for lung cancer invading the phrenic nerve because of technical limitations.

We report the first case of a patient in whom respiratory function was successfully preserved after performing these thoracoscopic one-stage procedures for T3 lung cancer invading the phrenic nerve.

## Case presentation

**Fig. 1:**
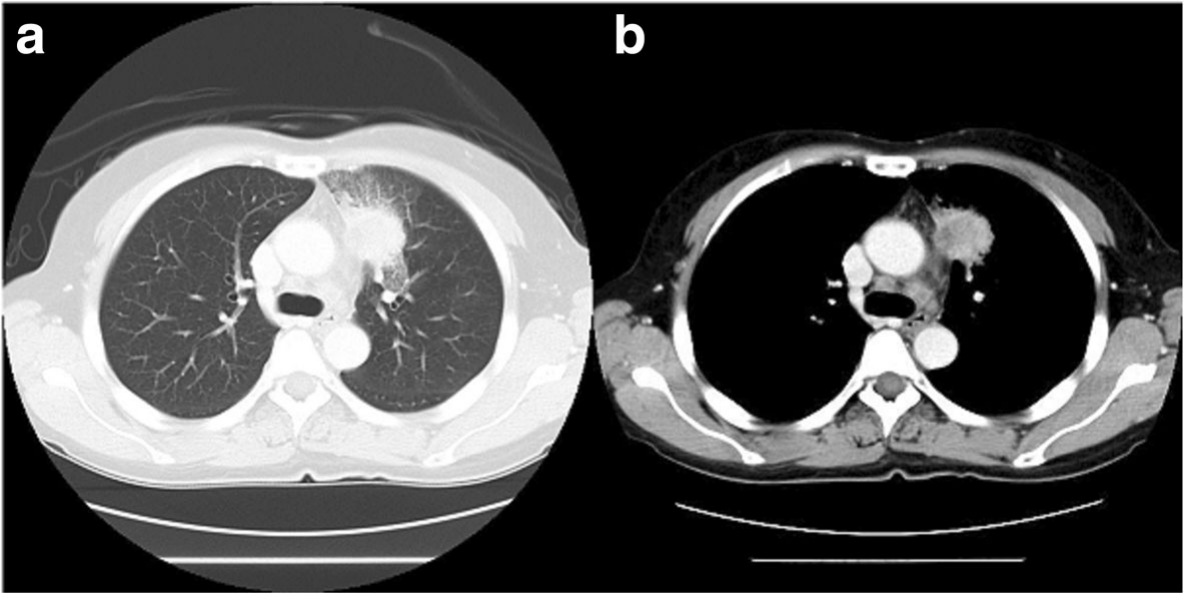
Preoperative computed tomography scan showing a 35 × 28-mm tumour in the anterior segment (segment 3) of the left upper lobe (**a**) and the anterior mediastinal fat tissue invaded by the tumour (**b**)

A 56-year-old woman was referred to our hospital for surgical treatment of a T3N0M0 primary adenosquamous carcinoma measuring 35 × 28 mm in the anterior segment (segment 3) of the left upper lobe without mediastinal lymph node swelling in preoperative computed tomography. The ppo VC and ppo FEV1 were 2.68 L and 2.22 L, respectively. The preoperative computed tomography scan revealed that the tumour had invaded the anterior mediastinal fat tissue and phrenic nerve (Fig. 1a, b).

**Fig. 2:**
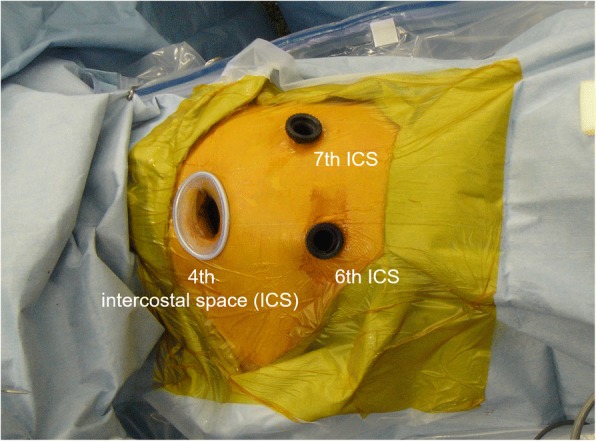
Port placements

**Fig. 3:**
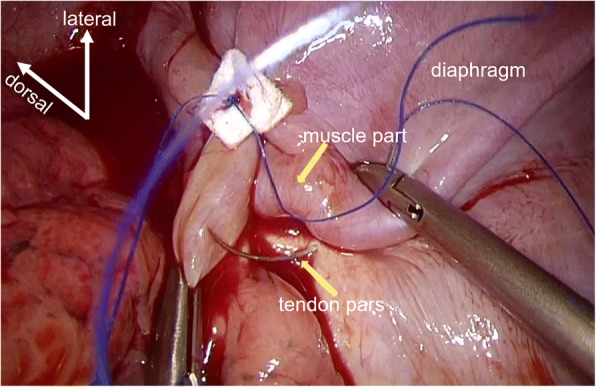
Thoracoscopic view during thoracoscopic diaphragmatic plication

**Fig. 4:**
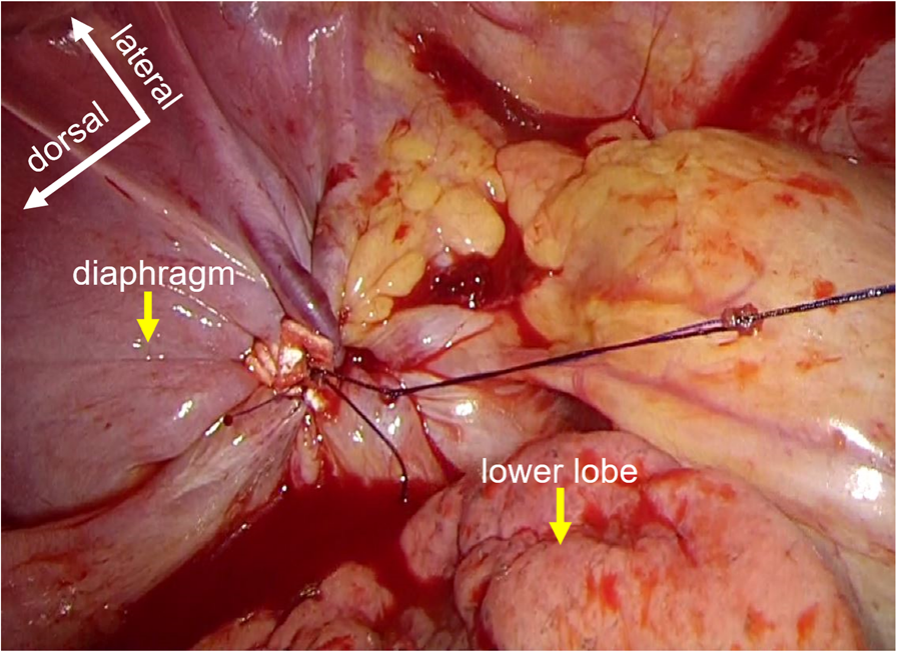
Thoracoscopic view after diaphragmatic plication

The patient was placed in a lateral position on the operating table under general anaesthesia with selective lung ventilation. Two thoracoport trocars (15 mm) were placed in the sixth intercostal space (ICS) at the anterior axillary line and in the seventh ICS at the posterior axillary line. An anterolateral mini-thoracotomy (35 mm) was performed in the fourth ICS for left upper lobectomy (Fig. 2). We resected the phrenic nerve and pericardial fat with an optimal surgical margin and then performed left upper lobectomy and lymph node dissection. Thoracoscopic diaphragmatic plication was performed with 3–0 Prolene sutures running from the dorsolateral to ventromedial diaphragm in order to oversew the diaphragmatic tendon pars and imbricate the muscle part (Fig. 3). Dacron pledgets were only used for the first suture and the suture was retracted to the cranial side during needle stitch (Fig. 4). The thoracoscope was placed through the thoracoport trocar in the seventh ICS at the posterior axillary line and the plication was performed through the thoracoport trocar in the sixth ICS at the anterior axillary line with an endoscopic needle holder.

**Fig. 5:**
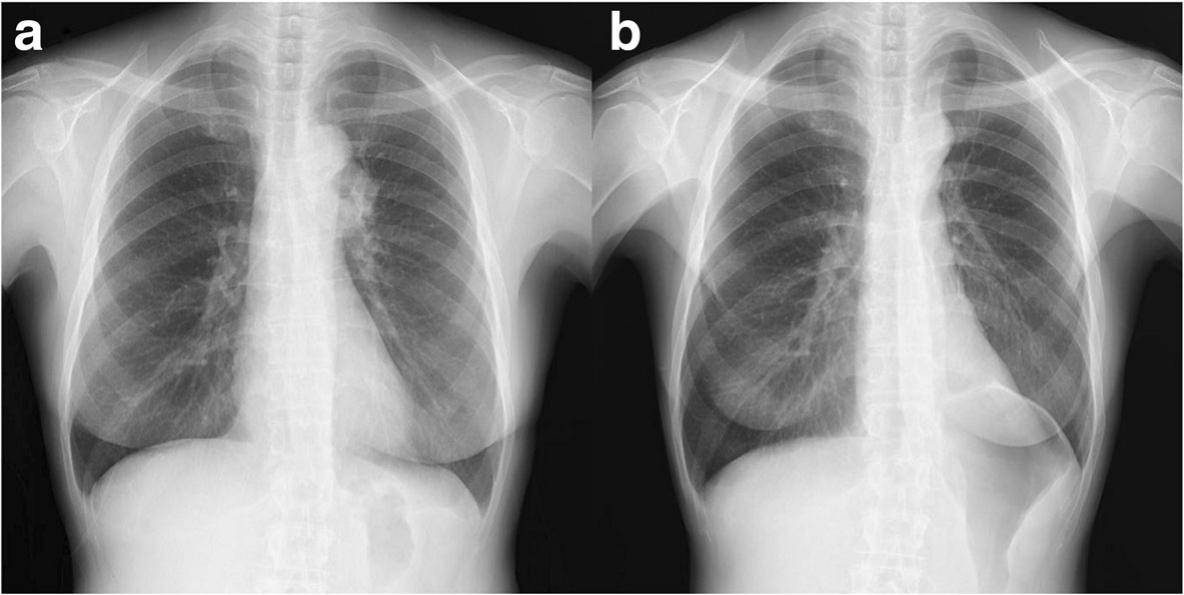
**a** Preoperative chest radiograph and **b** postoperative chest radiograph at 1 year showing mild elevation of the left hemidiaphragm

The pathological diagnosis was a T3N2M0 primary adenosquamous carcinoma invading the phrenic nerve with negative surgical margins. The patient’s postoperative course was favourable without any complications. No clinical symptoms were observed during the follow-up. Pulmonary function testing performed 1 year after the surgery revealed VC and FEV1 values of 2.36 L and 2.08 L, respectively. The representative chest radiographs of the left hemidiaphragm showed a normal position preoperatively and only mild elevation postoperatively (Fig. 5a, b).

## Discussion and conclusions

Diaphragmatic paralysis decreases VC by 20 to 30% and leads to pulmonary complications [1]. Although this does not generally cause respiratory difficulty in healthy adults, the risk of respiratory difficulty is high in most patients with lung cancer because they tend to be older and might have a history of heavy smoking inducing low pulmonary function. Tokunaga et al. reported that after surgical plication for diaphragmatic paralysis during extended surgery for pulmonary cancer, mediastinal tumour, and malignant pleural mesothelioma, the mean ratio of measured postoperative VC to ppo VC was 88.2% and the mean ratio of measured postoperative FEV1 to ppo FEV1 was 98.5% [2]. In the present case, the ratios of the actual postoperative VC to ppo VC and actual postoperative FEV1 to ppo FEV1 were 88 and 94%, respectively, indicating that respiratory function was preserved postoperatively. The patient did not experience any clinical symptoms 1 year postoperatively.

Previous research studies have reported on phrenic nerve reconstruction for diaphragmatic paralysis [3, 4]. Respiratory function for FEV1 in patients undergoing reconstruction of the phrenic nerve and diaphragmatic plication improved by averages of 13 and 17%, respectively. In addition, forced VC improved by an average of 14% after reconstruction of the phrenic nerve and 17% after diaphragmatic plication [3]. In our case, it was difficult to make a preoperative decision about the extent of phrenic nerve resection. Our decision to perform diaphragmatic plication was influenced by an increased likelihood that the patient would maintain her respiratory function.

Consensus was reached about performing early plication for diaphragmatic paralysis in infants and young children [5]. Çelik et al. suggested that early and timely plication for diaphragmatic paralysis improves functional status and shortens the length of hospitalization [6]. However, the timing of surgical plication remains controversial in adults. Diaphragmatic plication for phrenic nerve paralysis after surgery has been considered as a second surgery because nerve function may recover [7]. Preoperative pulmonary function testing and evaluation of phrenic nerve activity by electromyography can be performed for selecting patients in need of surgical treatment before surgical plication in two stages. However, two-stage diaphragmatic plication after ipsilateral thoracic surgery is more difficult owing to pleural adhesions, especially following lower lobectomy and posterior mediastinal lymph node dissection. In the present case, the recovery of the nerve could not be expected because nerve resection without reconstruction was performed. Therefore, a one-stage procedure of diaphragmatic plication was performed, and the patient experienced no perioperative complications.

One-stage pulmonary lobectomy and diaphragmatic plication have been reported for lung cancer in thoracotomy but not in thoracoscopic surgery. Thoracotomy transiently reduces diaphragmatic function. Demos et al. favour performing plication in a minimally invasive manner, as this deficit recovers more quickly after thoracoscopic surgery than after thoracotomy [8]. However, suturing seems to be the primary problem of diaphragmatic plication in thoracoscopic surgery, and splenic injury and/or greater omentum injury caused by blind suturing may result in abdominal bleeding. We changed the operating table to a reversed Trendelenburg position, with the patient in a lateral position during diaphragmatic plication. Furthermore, the diaphragm was sutured while staying away from the dorsal depth so as not to injure the spleen during the left side procedure.

Thoracoscopic one-stage lobectomy with phrenic nerve resection and diaphragmatic plication as a one-stage procedure is effective for preserving postoperative pulmonary function and reduces the necessity for pleurolysis due to pleural adhesion and the risks associated with additional general anaesthesia.

## Abbreviations

FEV1: Forced expiratory volume in 1 second
ICS: Intercostal space
ppo FEV1: Predicted postoperative FEV1
ppo VC: Predicted postoperative VC
VC: Vital capacity
